# Deciphering the Genetics of Primary Angioedema with Normal Levels of C1 Inhibitor

**DOI:** 10.3390/jcm9113402

**Published:** 2020-10-23

**Authors:** Gedeon Loules, Faidra Parsopoulou, Maria Zamanakou, Dorottya Csuka, Maria Bova, Teresa González-Quevedo, Fotis Psarros, Gregor Porebski, Matthaios Speletas, Davide Firinu, Stefano del Giacco, Chiara Suffritti, Michael Makris, Sofia Vatsiou, Andrea Zanichelli, Henriette Farkas, Anastasios E. Germenis

**Affiliations:** 1 CeMIA SA, Makriyianni 31, GR-41334 Larissa, Greece; gedloules@cemia.eu (G.L.); fparsopoulou@cemia.eu (F.P.); mzamanakou@cemia.eu (M.Z.); svatsiou@cemia.eu (S.V.); 2 Department of Immunology & Histocompatibility, School of Health Sciences, Faculty of Medicine, University of Thessaly, Panepistimiou 3, GR-41500 Biopolis, Larissa, Greece; maspel@med.uth.gr; 3 Hungarian Angioedema Center, 3rd Department of Internal Medicine, Semmelweis University, Kutvolgyi ut 4, H-1125 Budapest, Hungary; csuka.dorottya@med.semmelweis-univ.hu (D.C.); farkas.henriette@med.semmelweis-univ.hu (H.F.); 4 Department of Translational Medicine, University of Naples, Via S. Pansini 5, 80131 Naples, Italy; bovamaria@virgilio.it; 5 Reference Unit for Angioedema in Andalusia, Allergy Department, Virgen del Rocío University Hospital, Av Manuel Siurot s/n, 41013 Seville, Spain; mtgonzalezq@gmail.com; 6 Department of Allergology, Navy Hospital, Deinokratous 70, GR-11521 Athens, Greece; psarros@allergy.gr; 7 Department of Clinical and Environmental Allergology, Jagiellonian University Medical College, Sniadeckich 10, 31-531 Krakow, Poland; g.porebski@uj.edu.pl; 8 Department of Medical Sciences and Public Health, University of Cagliari, 09042 Monserrato, Italy; davide.firinu@unica.it (D.F.); delgiac@gmail.com (S.d.G.); 9 Department of Biomedical and Clinical Sciences Luigi Sacco, University of Milan, Via G.B. Grassi 74, 20157 Milan, Italy; chiarasuffritti@gmail.com (C.S.); andrea.zanichelli@unimi.it (A.Z.); 10 Allergy Unit, 2nd Department of Dermatology and Venereology, National and Kapodistrian University of Athens, University Hospital “Attikon”, Rimini 1, GR-12462 Chaidari, Athens, Greece; mmakris.allergy@gmail.com

**Keywords:** next-generation sequencing, pedigree analysis, primary angioedema, primary angioedema with normal C1 inhibitor

## Abstract

The genetic alteration underlying the great majority of primary angioedema with normal C1 inhibitor (nl-C1-INH-HAE) cases remains unknown. To search for variants associated with nl-C1-INH-HAE, we genotyped 133 unrelated nl-C1-INH-HAE patients using a custom next-generation sequencing platform targeting 55 genes possibly involved in angioedema pathogenesis. Patients already diagnosed with *F12* alterations as well as those with histaminergic acquired angioedema were excluded. A variant pathogenicity curation strategy was followed, including a comparison of the results with those of genotyping 169 patients with hereditary angioedema due to C1-inhibitor deficiency (C1-INH-HAE), and only filtered-in variants were studied further. Among the examined nl-C1-INH-HAE patients, carriers of neither the *ANGPT1* p.Ala119Ser nor the *KNG1* p.Met379Lys variant were found, whereas the *PLG* p.Lys330Glu was detected in four (3%) unrelated probands (one homozygote). In total, 182 different variants were curated, 21 of which represented novel mutations. Although the frequency of variants per gene was comparable between nl-C1-INH-HAE and C1-INH-HAE, variants of the *KNG1* and *XPNPEP1* genes were detected only in nl-C1-INH-HAE patients (six and three, respectively). Twenty-seven filtered variants in 23 different genes were detected in nl-C1-INH-HAE more than once, whereas 69/133 nl-C1-INH-HAE patients had compound heterozygotes of filtered variants located in the same or different genes. Pedigree analysis was performed where feasible. Our results indicate the role that alterations in some genes, like *KNG1*, may play in disease pathogenesis, the complex trait that is possibly underlying in some cases, and the existence of hitherto unrecognized disease endotypes.

## 1. Introduction

Primary angioedema is defined as localized and self-limiting edema of the subcutaneous and submucosal tissue occurring in the absence of wheals and of a causative factor. According to the criteria of the Hereditary Angioedema International Working Group [1], all hereditary forms as well as the two idiopathic forms of acquired angioedema (histaminergic and non-histaminergic) can be considered as primary angioedema. Hereditary angioedema due to C1-INH deficiency (C1-INH-HAE), the prototype of primary angioedema, is an autosomal dominant disease caused by deleterious mutations in the *SERPING1* gene, leading to quantitative and/or functional C1 inhibitor (C1-INH) deficiency [2]. Normal C1-INH levels and function characterize all other forms of primary angioedema, which clinically present with individual attacks indistinguishable from C1-INH-HAE attacks, despite differing from C1-INH-HAE in many aspects [3]. Until recently, the only genetic defects known to be associated with the hereditary forms of primary angioedema with normal C1-INH levels were mutations in the *F12* gene [4,5]. All other familial cases of angioedema with normal C1-INH levels were characterized as unknown angioedema. In 2019, next-generation sequencing technologies provided new insights into the genetics of primary angioedema with normal C1 inhibitor (nl-C1-INH-HAE). Two new missense mutations in *ANGPT1* (c.807G>T, p.Ala119Ser) and *PLG* (c.988A>G, p.Lys330Glu) genes were detected in association with the disease, whereas family segregation and meticulous functional studies have proved their pathogenicity [6,7,8]. Recently, Bork et al. [9] reported a hitherto unknown variant in exon 10 of the *KNG1* gene (c.1136T>A, p.Met379Lys) co-segregated with clinical symptoms of hereditary angioedema (HAE) with normal C1-INH levels in three generations of a large German family.

Interestingly, the recently discovered pathogenic variants expanded our concept of nl-C1-INH-HAE pathophysiology beyond the contact system indicating new disease endotypes [6,10,11]. Moreover, a series of patients misdiagnosed as idiopathic non-histaminergic acquired angioedema (InH-AAE) have been reported in the literature, who, after genotyping, were proved to be suffering nl-C1-INH-HAE associated with *F12, PLG*, or *ANGPT1* mutations [12]. Thus, further uncovering the genetic basis of nl-C1-INH-HAE is expected not only to facilitate a better understanding of disease pathophysiology that could drive the discovery of new therapeutic targets but also to provide useful indicators for the clinical management of the disease. To this aim, here, we applied a custom next-generation sequencing (NGS) platform targeting a series of genes entangled in the metabolism and function of bradykinin to detect candidate genes involved in the pathogenesis of nl-C1-INH-HAE.

## 2. Experimental Section

### 2.1. Patients

Patients fulfilling the diagnostic criteria of primary angioedema according to the Hereditary Angioedema International Working Group [1] and presenting with normal C1-INH plasma levels were included in the study. Beyond those diagnosed with hereditary angioedema with normal C1-INH, patients with idiopathic non-histaminergic acquired angioedema were included in this group, since they represent a temporary exclusion diagnosis that does not rule out either the appearance of angioedema in the next generation or the presence of a yet unidentified genetic background [12]. Patients already diagnosed with hereditary angioedema with normal C1-INH and factor XII mutation (FXII-HAE), as well as those with histaminergic acquired angioedema, were excluded.

In total, 133 unrelated patients (53 Hungarian, 32 Italian, 27 Spanish, 12 Greek, and 9 Polish) (35 male; age 40.8 ± 17.4 years) were enrolled in the study. Their mean (±SD) age at disease onset was 27.0 ± 16.4 years (median: 24 years). One in 133 patients had suffered only one angioedema attack during their life; the other 132/133 had a mean frequency of angioedema attacks of 7.8 per year (median: 5 per year). Of the 133 patients, 104 presented with a family history of angioedema, whereas 31/133 patients were on long-term prophylaxis with tranexamic acid. A further 169 patients with C1-INH-HAE were genotyped as controls to search for the presence of variants common in the two forms of angioedema that possibly affect the clinical expression of the disease.

The study was carried out according to the principles of good clinical practice and adhered to the ethical standards of the Declaration of Helsinki with written informed consent from all subjects. The Ethics Committee of the University of Thessaly approved the protocol of the study.

### 2.2. Genotyping

A custom NGS panel was designed using the Ion AmpliSeq Thermo Fisher Scientific Designer (Thermo Scientific, Waltham, Massachusetts, US) to analyze 55 genes (all coding regions and exon–intron splice junctions) (Supporting Information, Table S1) possibly involved in angioedema pathogenesis and/or the clinical phenotype. The gene list was compiled from literature data on angioedema and genetic predisposition, protein–protein interaction networks, and pathway analysis. In total, 825 amplicons in two primer pools provide 99.61% coverage of all targeted regions.

To construct DNA libraries for each sample using the Ion AmpliSeq Library Kit 2.0 (Thermo Scientific, Waltham, MA, USA), 10 ng of gDNA per primer pool was used. The produced libraries were indexed with a unique adapter using the Ion Xpress barcode adapter kit (Thermo Scientific, Waltham, MA, USA). Barcoded libraries were purified using the Agencourt AMPure XP Beads (Beckman Coulter, Brea, CA, USA), quantified with a Qubit 2.0 fluorometer (Thermo Scientific, Waltham, MA, USA), diluted to 100 pM and pooled in equimolar proportion. Template preparation, enrichment, and chip loading were carried out on the Ion Chef system (Thermo Scientific, Waltham, MA, USA). Sequencing was performed on S5XL on 520 and 530 chips, using the Ion 510, Ion 520, and Ion 530 Kit - Chef (Thermo Scientific, Waltham, MA, USA). All procedures were performed according to the manufacturer’s instructions.

Base calling, demultiplexing, and alignment to the hg19 reference genome (GRCh37) of the raw sequencing data were performed in Torrent Suite 5.10 software (Thermo Scientific, Waltham, MA, USA) using the default parameters. Variant calling was performed by the VariantCaller v.5.8.0.19 plug-in and coverage analysis by the CoverageAnalysis v.5.8.0.8 plug-in in Torrent Suite 5.10.

Confirmatory Sanger sequencing was appropriately performed where necessary. Since the causative *ANGPT1* variant (c.807G>T) had not been described at the time of the design of our NGS panel, this gene was not included among those analyzed by this method. Thus, *ANGPT1* genotyping was performed by Sanger sequencing as previously described [6].

### 2.3. Variant Pathogenicity Curation

All variants detected after alignment to the hg19 genome using the VariantCaller plug-in were annotated in Ion Reporter software v.5.6 (Thermo Scientific, Waltham, MA, USA) with the gene name and for their possible presence in the Single Nucleotide Polymorphism Database (v135) [13], the Exome Aggregation Consortium (ExAC) [14], the 1000 Genomes project [15], and the ClinVar [16] according to the recommendations of the Human Genome Variation Society (HGVS) [17]. SIFT [18] and PolyPhen version 2 [19] bioinformatics tools were used for in silico pathogenicity prediction of the variants. Alignments and all obtained sequences were visually inspected using the Integrative Genomics Viewer (IGV) v.2.2 (Broad Institute, Cambridge, MA, USA).

Variants with a worldwide frequency of >1% (1000 Genomes Global Minor Allele Frequency, ExAC) and polymorphisms for which no disease associations are reported in the ClinVar database, as well as synonymous and intronic single-nucleotide variants (SNVs), were excluded from further analysis.

## 3. Results and Discussion

Among the nl-C1-INH-HAE patients, no carriers of the *ANGPT1* p.Ala119Ser variant were found, indicating that at least this mutation of the *ANGPT1* gene represents very rare causative genetic damage. However, the *PLG* p.Lys330Glu variant was detected in four (3%) unrelated probands (one homozygote), which have already been described in detail elsewhere [20]. Pedigree analysis of these cases confirmed the incomplete penetrance of this alteration. Including our cases, more than 100 patients with nl-C1-INH-HAE due to this mutation have been reported in the literature since its first description [21].

Among the variants identified in the 55 analyzed genes, 182 different variants were filtered in and included in further analysis. Twelve alterations occurred in the 5′ untranslated region (UTR) (6.6%) and 18 in the 3′-UTR (10%). Missense mutations corresponded to 76.6% of the total, followed by small insertions/deletions leading to frameshift (1%), non-sense (3.3%), splice site (0.5%), stop-loss (0.5%), and non-frameshift insertions/deletions (1.5%). A list containing all variants is found in Table S2 (Supporting Information). Of the 182 mutations (indicated in black in Table S2), 21 were not previously reported in population databases (novel mutations). The frequency of variants per gene was not significantly different between nl-C1-INH-HAE and C1-INH-HAE patients, with the exception of *KNG1* and *XPNPEP1* genes, where six and three variants were detected, respectively, in the nl-C1-INH-HAE group but none in the C1-INH-HAE group.

A series of 27 filtered variants in 23 different genes was detected in our material more than once. As shown in Table 1, in a proportion of these variants, their allele frequencies among nl-C1-INH-HAE patients were significantly different from those in the European population or even in our C1-INH-HAE cohort. According to the guidelines of the American College of Medical Genetics and Genomics [22], this is a criterion in favor of the pathogenicity of the variants. The exact contribution of each one of these variants in the pathogenesis or in the clinical phenotype of the disease is difficult to envisage. However, a finding that merits particular attention is the frequency of filtered androgen receptor gene (*AR*) variants, which, among nl-C1-INH-HAE patients, is significantly higher than that in both the European population and in the cohort of C1-INH-HAE controls. Further studies are worth undertaking to investigate the possible correlation of these variants with the estrogen-dependence of the disease’s clinical phenotype.

Sixty-nine of the examined nl-C1-INH-HAE patients were heterozygous for more than one and up to nine filtered variants located in the same or different genes (compound heterozygotes). No correlation was found between the number of heterozygous variants carried by patients and their age at disease onset or the frequency of attacks.

Family segregation studies were performed when feasible and provided useful information. Firstly, the variants p.Leu140Val and p.Ala177Val of the *F12* gene proved to be non-pathogenic. However, the novel *PLG* p.Val728Glu (c.2183T>A) variant was found to co-segregate with angioedema symptoms in a Greek patient (male, 15 years old) and their suffering father (52 years old) but not in his unaffected mother. His 10-year-old sister also carries the variant but she has not demonstrated disease symptoms as yet. The p.Val728Glu variant is located inside the plasmin serine protease domain (residues 562–791), which is an active serine protease with a wide substrate specificity [23]. Thus, the p.Val728Glu substitution could eventually affect functional interrelationships between the plasminogen/plasmin system and the kinin pathway, leading to an alteration in vasopermeability.

**Figure 1:**
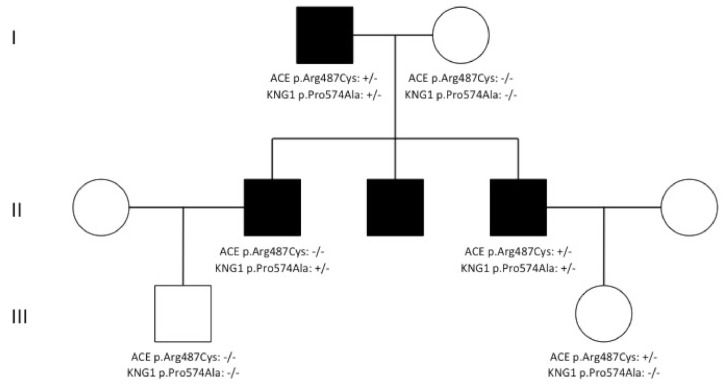
Pedigree demonstrating co-segregation of the missense mutation of the *KNG1* p.Pro574Ala (c.1720C>G) and the *ACE* p.Arg487Cys (c.1459C>T) variants with nl-C1-INH-HAE.

The recently reported *KNG1* p.Met379Lys variant [9] was not detected in any of our nl-C1-INH-HAE patients. However, the *KNG1* p.Pro574Ala (c.1720C>G) variant was detected in three affected members (two brothers and their father) of an Italian family but not in three asymptomatic relatives. Two of the patients suffered typical disease with repeated angioedema attacks, whereas the third had only experienced one attack during his life following a viral infection. Interestingly, the two patients who suffered repeated attacks were also carriers of the *ACE* p.Arg487Cys (c.1459C>T) variant. The same variant, despite it being predicted as deleterious by bioinformatics tools, was also detected in one of the three analyzed asymptomatic relatives (Figure 1). It seems that the *KNG1* p.Pro574Ala variant presents with incomplete penetrance or that its possible pathogenicity depends upon its compound heterozygosity with the *ACE* p.Arg487Cys variant. In conjunction with the abovementioned high frequency of filtered *KNG1* variants observed among nl-C1-INH-HAE patients, these findings indicate that variations in the *KNG1* gene could contribute to the pathogenesis of the disease; thus, they deserve further consideration.

The genes encoding for tryptases (*TPSAB1, TPSD1*, and *TPSG1*) were included in the panel of analyzed genes because raised serum tryptase has been occasionally observed in cases of acquired angioedema [24,25]. The variant p.Arg158Gln (c.473G>A) of the *TPSG1* gene was detected in all three affected women in three generations and in one of the three examined asymptomatic first-degree relatives of an Italian family. Should this finding be confirmed by further studies, it would implicate new pathways or cells (e.g., mastocytes) in the pathogenesis of nl-C1-INH-HAE.

A final remarkable finding was that the two suffering members (a mother and her daughter) of a Hungarian family were carriers of the same series of novel or rare variants in different genes: *BDKRB1* p.Arg282Ter, *CPN1* p.Glu407Lys, *SERPING1* c.*57C>G (3′UTR), *PLAUR* p.Met268Val, *MASP1* p.Val680Ala, *TLR4* p.Cys281Tyr, and MPO p.Arg524His. This observation suggests that, at least in certain cases, nl-C1-INH-HAE could be the result of the cumulative effect of multiple gene variations.

Taken together, the above observations clearly demonstrate that, genetically, nl-C1-INH-HAE is an extremely complex disorder. The relatively small size of the examined cohort of patients and the non-availability of families for pedigree analysis represent the main limitations of the study, which is true for all rare disease studies. Thus, strong evidence of the causative effect of certain variants has not been provided. Nevertheless, the results of the study helpfully highlight the role that alterations in some genes, like *KNG1,* may play in the pathogenesis of the disease, the complex trait that is possibly underlying some cases, and the existence of hitherto unrecognized disease endotypes. Finally, it must be underlined that every day, contemporary genomic approaches discover new genes associated with the disease, indicating the involvement of new pathways in its pathogenesis (e.g., the *ANGPT1* [6] and the *MYOF* [26] genes). Therefore, beyond the genes examined in this study, there are many other candidate disease genes remaining to be examined, like many endothelium-associated ones.

## Figures and Tables

**Table 1:** Variants with a worldwide allelic frequency of <1% that were detected in our material more than once. EMAF: European minor allele frequency; ExAC ENFAF: ExAC European non-Finnish allele frequency; nl-C1-INH-HAE AF: allele frequency among nl-C1-INH-HAE patients; C1-INH-HAE AF: allele frequency among C1-INH-HAE patients, P1: EMAF vs. nl-C1-INH-HAE AF, P2: EMAF vs. C1-INH-HAE AF, P3: ni-C1-INH-HAE AF vs. C1-INH-HAE AF.

Genes	Coding	Amino Acid Change	dbSNP	SIFT	PolyPhen	EMAF	ExAC ENFAF	nl-C1-INH-HAE AF	C1-INH-HAE AF	P1	P2	P3
*BDKRB1*	c.721G>A	p.Gly241Arg	rs45528332	tolerated	probably damaging	0.0037	0.0052	0.0113	0.0148	0.1500	0.0350	0.7000
*MME*	c.674G>C	p.Gly225Ala	rs147564881	tolerated	probably damaging	0.0023	0.0033	0.0113	0.0030	0.0310	0.7400	0.2100
*PLAUR*	c.802A>G	p.Met268Val	rs138492321	tolerated	possibly damaging	0.0062	0.0045	0.0188	0.0000	0.0440	0.1500	0.0110
*C1S*	c.943G>A	p.Asp315Asn	rs117907409	deleterious	probably damaging	0.0053	0.0052	0.0113	0.0059	0.2400	0.8300	0.4700
*F13B*	c.1025T>C	p.Ile342Thr	rs17514281	deleterious	possibly damaging	0.0097	0.0098	0.0263	0.0059	0.0380	0.4900	0.0390
*F2*	c.*97G>A		rs1799963			0.0080		0.0263	0.0148	0.0130	0.2600	0.3100
*TLR4*	c.842G>A	p.Cys281Tyr	rs137853920	deleterious	probably damaging	0.0044	0.0027	0.0150	0.0030	0.0420	0.7900	0.1000
*KRT1*	c.1669A>G	p.Ser557Gly	rs77846840	tolerated	benign		0.0019	0.0263	0.0296			0.8000
*SERPINE1*	c.*180C>T		rs41334349			0.0110		0.0226	0.0266	0.1400	0.0400	0.7500
*AR*	c.-207C>A		rs189146053			0.0000		0.0188	0.0030	<0.0001	0.0844	0.0500
*AR*	c.1174C>T	p.Pro392Ser	rs201934623	tolerated	benign	0.0000	0.0041	0.0113	0.0000	0.0007		0.0500
*TPSAB1*	c.407A>G	p.His136Arg	rs201820654	tolerated	benign		0.0034	0.0113	0.0089			0.7600
*TPSG1*	c.508G>A	p.Gly170Arg	rs117769620	tolerated	benign	0.0065	0.0073	0.0188	0.0118	0.0757	0.3893	0.4832
*ELANE*	c.770C>T	p.Pro257Leu	rs17216663	tolerated	benign	0.0108	0.0080	0.0188	0.0030	0.3062	0.1775	0.0530
*F12*	c.418C>G	p.Leu140Val	rs35515200	tolerated	possibly damaging	0.0042	0.0033	0.0075	0.0030	0.4533	0.7904	0.4287
*F12*	c.530C>T	p.Ala177Val	rs144821595	tolerated	benign	0.0002	0.0001	0.0075	0.0000	<0.0001	0.7948	0.1103
*ACE*	c.1453C>G	p.Pro485Ala	rs202178737	deleterious	benign	0.0000	0.0001	0.0075	0.0000	0.0059		0.1103
*BDKRB1*	c.844C>T	p.Arg282Ter	rs145322761			0.0035	0.0038	0.0075	0.0030	0.4533	0.7904	0.4287
*PLG*	c.266G>A	p.Arg89Lys	rs143079629	tolerated	benign	0.0100	0.0108	0.0075	0.0030	0.7164	0.2177	0.4287
*KLK3*	c.629C>G	p.Ser210Trp	rs61729813	deleterious	probably damaging	0.0110	0.0109	0.0075	0.0178	0.6223	0.3319	0.2748
*DPP4*	c.796G>A	p.Val266Ile	rs56179129	tolerated	benign	0.0060	0.0045	0.0075	0.0000	0.7755	0.1547	0.1103
*PLAU*	c.1048T>C	p.Tyr350His	rs72816325	deleterious	probably damaging	0.0058	0.0059	0.0075	0.0000	0.7755	0.1547	0.1103
*PLAUR*	c.-87C>T		rs147665588			0.0060		0.0075	0.0089	0.7755	0.5702	0.8550
*F13A1*	c.1730C>T	p.Thr577Met	rs143711562	tolerated	benign	0.0029	0.0020	0.0075	0.0000	0.2930	0.3149	0.1103
*TNF*	c.251C>T	p.Pro84Leu	rs4645843	tolerated	benign	0.0030	0.0028	0.0075	0.0030	0.2930	0.9945	0.4287
*GPER1*	c.14C>T	p.Ser5Phe	rs117290655	tolerated	benign	0.0048	0.0045	0.0075	0.0089	0.6173	0.4193	0.8550
*MPO*	c.2031-2A>C		rs35897051			0.0072	0.0071	0.0075	0.0059	0.9227	0.8391	0.8096

## Supplementary Materials

The following are available online at https://www.mdpi.com/2077-0383/9/11/3402/s1: Table S1: The genes analyzed by our custom NGS panel and their coverages; Table S2: The filtered-in variants detected among nl-C1-INH-HAE patients. Variants not previously reported in population databases (novel mutations) are indicated in bold.
